# Supplementary material for: Preparation and in vivo effectiveness evaluation of heparin-loaded PLGA@PCL core-shell fiber small-diameter vascular grafts
Source: PLoS One. 2026 Jan 16;21(1):e0337192. doi: 10.1371/journal.pone.0337192 (PMC12810837; doi:10.1371/journal.pone.0337192)
Supplement: Attachment — Submitted filename: Response to Reviewers.docx [file pone.0337192.s002.docx]

Dear Reviewers，

Thank you for your insightful comments and suggestions on our manuscript. We appreciate the time you have taken to provide feedback, and we have carefully considered each of your points. Our responses are provided below:

**Journal Requirements:**

1. When submitting your revision, we need you to address these additional requirements.

Please ensure that your manuscript meets PLOS ONE's style requirements, including those for file naming. The PLOS ONE style templates can be found at 
<https://journals.plos.org/plosone/s/file?id=wjVg/PLOSOne_formatting_sample_main_body.pdf> and 
<https://journals.plos.org/plosone/s/file?id=ba62/PLOSOne_formatting_sample_title_authors_affiliations.pdf>.

**Response: We have modified this paper to meet PLOS ONE's style requirements.**

2. To comply with PLOS ONE submissions requirements, in your Methods section, please provide additional information regarding the experiments involving animals and ensure you have included details on (1) methods of sacrifice, (2) methods of anesthesia and/or analgesia, and (3) efforts to alleviate suffering.

**Response: The revised version has included these contents.**

3. We noticed you have some minor occurrence of overlapping text with the following previous publication(s), which needs to be addressed:

Fabrication and performance evaluation of PLCL-hCOLIII small-diameter vascular grafts crosslinked with procyanidins
Author links open overlay panel - <https://doi.org/10.1016/j.ijbiomac.2023.126293>

Remodeling of structurally reinforced (TPU+PCL/PCL)-Hep electrospun small-diameter bilayer vascular grafts interposed in rat abdominal aortas - <https://doi.org/10.1039/D1BM01653A>

(Among others)

In your revision ensure you cite all your sources (including your own works), and quote or rephrase any duplicated text outside the methods section. Further consideration is dependent on these concerns being addressed.

**Response: We have made modifications to reduce the overlap with our published papers.**

4. Thank you for stating the following financial disclosure: 
 [This work was supported by the National Key R&D Program of China (No.2022YFC2409802) and the National Key R&D Program of China (2017YFC1104101).].  
Please state what role the funders took in the study. If the funders had no role, please state: ""The funders had no role in study design, data collection and analysis, decision to publish, or preparation of the manuscript."" 
If this statement is not correct you must amend it as needed. 
Please include this amended Role of Funder statement in your cover letter; we will change the online submission form on your behalf.

**Response: We have made modifications in the manuscript and the cover letter.**

5. Thank you for stating the following in the Acknowledgments Section of your manuscript: 
[This work was supported by the National Key R&D Program of China (No.2022YFC2409802) and the National Key R&D Program of China (2017YFC1104101).]
We note that you have provided funding information that is not currently declared in your Funding Statement. However, funding information should not appear in the Acknowledgments section or other areas of your manuscript. We will only publish funding information present in the Funding Statement section of the online submission form. 
Please remove any funding-related text from the manuscript and let us know how you would like to update your Funding Statement. Currently, your Funding Statement reads as follows:  [This work was supported by the National Key R&D Program of China (No.2022YFC2409802) and the National Key R&D Program of China (2017YFC1104101).]

Please include your amended statements within your cover letter; we will change the online submission form on your behalf.

**Response: We have made modifications in the manuscript and the cover letter.**

6. In the online submission form, you indicated that [Data can be obtained by emailing the corresponding author of this article.]. 
All PLOS journals now require all data underlying the findings described in their manuscript to be freely available to other researchers, either 1. In a public repository, 2. Within the manuscript itself, or 3. Uploaded as supplementary information.
This policy applies to all data except where public deposition would breach compliance with the protocol approved by your research ethics board. If your data cannot be made publicly available for ethical or legal reasons (e.g., public availability would compromise patient privacy), please explain your reasons on resubmission and your exemption request will be escalated for approval.

**Response: All data could be acquired within the manuscript itself.**

7. Please include your full ethics statement in the ‘Methods’ section of your manuscript file. In your statement, please include the full name of the IRB or ethics committee who approved or waived your study, as well as whether or not you obtained informed written or verbal consent. If consent was waived for your study, please include this information in your statement as well.

**Response: All experimental procedures were performed under institutional guidelines for animal care and approved by the Animal Ethics Committee of Fuwai Hospital Chinese Academy of Medical Sciences (Beijing, China).**

8. We note that Figure(s) 1, 3a and 4 in your submission contain copyrighted images. All PLOS content is published under the Creative Commons Attribution License (CC BY 4.0), which means that the manuscript, images, and Supporting Information files will be freely available online, and any third party is permitted to access, download, copy, distribute, and use these materials in any way, even commercially, with proper attribution. For more information, see our copyright guidelines: <http://journals.plos.org/plosone/s/licenses-and-copyright>.

We require you to either (1) present written permission from the copyright holder to publish these figures specifically under the CC BY 4.0 license, or (2) remove the figures from your submission:

a. You may seek permission from the original copyright holder of Figure(s) 1, 3a and 4 to publish the content specifically under the CC BY 4.0 license. 
 
We recommend that you contact the original copyright holder with the Content Permission Form (<http://journals.plos.org/plosone/s/file?id=7c09/content-permission-form.pdf>) and the following text:
“I request permission for the open-access journal PLOS ONE to publish XXX under the Creative Commons Attribution License (CCAL) CC BY 4.0 (<http://creativecommons.org/licenses/by/4.0/>). Please be aware that this license allows unrestricted use and distribution, even commercially, by third parties. Please reply and provide explicit written permission to publish XXX under a CC BY license and complete the attached form.”
 
Please upload the completed Content Permission Form or other proof of granted permissions as an ""Other"" file with your submission. 
 
In the figure caption of the copyrighted figure, please include the following text: “Reprinted from [ref] under a CC BY license, with permission from [name of publisher], original copyright [original copyright year].”
 
b. If you are unable to obtain permission from the original copyright holder to publish these figures under the CC BY 4.0 license or if the copyright holder’s requirements are incompatible with the CC BY 4.0 license, please either i) remove the figure or ii) supply a replacement figure that complies with the CC BY 4.0 license. Please check copyright information on all replacement figures and update the figure caption with source information. If applicable, please specify in the figure caption text when a figure is similar but not identical to the original image and is therefore for illustrative purposes only.

[Note: HTML markup is below. Please do not edit.]

Reviewers' comments:

Reviewer's Responses to Questions

 **Response: We believe that in the previous version, except picture 1, picture 3 and picture 4 do not involve copyright issues. In the newly revised version, we have also changed picture 1, so currently all images do not involve copyright issues.**

**Reviewer #1:**

1. The definition of Core-Shell fiber, which is the focus of this paper, and its structure should be explained clearly, including schemes.

 **Response:** **We have modified the part 2.2 in order to explain the core-shell structure clearly.**

2. There are no data evaluating the characteristics of Core-Shell fiber, so please add this.

 **Response:** **Thanks for your comment. In this paper, we also characterized the surface morphology and core-shell structure of core-shell fibers by SEM and TEM, and studied the mechanical properties and degradation properties of the vascular graft after surface heparinization. For the surface fixed heparin, toluidine blue method and APTT method were also used to determine the content and release of heparin in vitro. At the same time, the core-shell fiber surface is heparin-modified PCL, which has been investigated for cytocompatibility and toxicity in our previous work *(Hui X, et al. J Biomater Appl. 2020; 34 (6) : 812-826; Xiao Y, et al. Biomater Adv. 2024)*. In summary, as a small-caliber blood vessel, the characterization of the above properties can basically conclude that the core-shell fiber vascular graft in this submission can be used for carotid artery vascular transplantation in New Zealand white rabbits.**

3. In vivo experiments excluded the control experiment. If stable patency rates and tissue regeneration can be induced by blood vessels made of Core-shell fiber, this should be discussed in comparison with the results of other types of fibers, such as those with no core-shell fibers as a control.

 **Response:** **Thank you for your professional comments. We did conduct a long-term experiment of core-shell structure fiber and pure PCL fiber vascular graft in a rat model of abdominal aortic vascular transplantation and reported the corresponding experimental results *(Xiao Y, et al. Biomater Adv. 2024; 165-214018; Fang Z., et al., Biomater.Sci.10 (2022) 4257-4270)*, small diameter vascular grafts using core-shell structure fibers have shown higher vascular patency rates and better elastin remodeling in the same animal model. Therefore, on the basis of this experiment, the carotid artery of New Zealand white rabbits was transplanted. The results of the experiment were compared with those of the double-layer vascular grafts performed earlier by our research group *(Jin X, et al. Macromol Biosci. 2019; 19(8):e1900114; Hui X, et al. J Biomater Appl. 2020; 34(6):812-826)* and surface heparinized vascular grafts have shown better resistance to vascular aneurysm occurrence, elastin remodeling, and vascular patency. At the same time, due to animal ethical considerations, pure PCL fibrovascular was not set as a control.**

**Reviewer #2:**

1.Manuscript dosent have page and line no.

**Response: We have added page and line number in the revised version.**

2. In section 2.1 Materials - sentence "N-Hydroxy succinimide was purchased from Sigma (St. Louis, MO, USA) was purchased from was purchased from Innochem (Beijing, China)" looks incomplete.

**Response: We have completed this sentence.**

3. In the same section please provide Ethics approval no.

 **Response: The Ethics approval no. have been provided in the part of “Surgical procedures”.**

4. In section 2.2 Fabrication please clearly claim which polymer is forming core and which is forming shell

 **Response: We have modified the part 2.2 in order to explain the core-shell structure clearly.**

5. In section 2.3 authors used many short forms (EDC, DIPEA, HOBT, NHS, MES etc); it might be from their previous publication, please provide full form of the same in this manuscript

**Response: We have provided full form of these short forms in the revised version.**

6.In section 2.4 and 2.5 authors should include more characterization like DCS, XRD etc to improve understanding on thermal and crystalline behavior of the scaffolds

**Response: Thanks for your comment. In this paper, we also characterized the surface morphology and core-shell structure of core-shell fibers by SEM and TEM, and studied the mechanical properties and degradation properties of the vascular graft after surface heparinization. For the surface fixed heparin, toluidine blue method and APTT method were also used to determine the content and release of heparin in vitro. At the same time, the core-shell fiber surface is heparin-modified PCL, which has been investigated for cytocompatibility and toxicity in our previous work *(Hui X, et al. J Biomater Appl. 2020; 34 (6) : 812-826; Xiao Y, et al. Biomater Adv. 2024)*. In summary, as a small-caliber blood vessel, the characterization of the above properties can basically conclude that the core-shell fiber vascular graft in this submission can be used for carotid artery vascular transplantation in New Zealand white rabbits.**

7. In section 2.6 Why author used type I Collagenase for degradation of polymers?

**Response:** **Thanks a lot for this suggestion. This experiment was initially designed to investigate whether vascular grafts could be affected by matrix metalloproteinases during regeneration in vivo, and accordingly the more common type I collagenase was chosen as the active component of enzymatic degradation in vitro.**

8. in the same section "wt is the mass before degradation after accelerated degradation for 7 days" looks incomplete

**Response:** **We have revised this sentence.**

9.Section 2.8 Please check "werre was..")

**Response:** **We have revised this sentence.**

10. Section 2.9.1 authors mentioned that they kept SDVG in 75% alcohol for 20 min. have they studied the effect of alcohol on HCS? please compare it using FTIR

**Response:** **We are very sorry that due to the existing experimental conditions and the limitation of raw materials, we cannot re-prepare the material and carry out this test at present. But based on our previous experience, alcohol immersion has no effect on the material structure.**

11. Figure 1 Please provide the cross section of core-shell fiber

**Response: We are very sorry that due to the existing experimental conditions and the limitation of raw materials, we cannot re-prepare the material and recapture the picture of the materials.**

12. Figure 4 (C-D) it should be (C-F)

**Response: We have revised this sentence.**

13. Just before figure 6 authors mentioned (Figure 8); there is no figure 8 in the manuscript

**Response: We have revised it in the** **paragraph.**

14. In discussion please provide full form of VSMC

**Response:** **we have provided the full name of VSMCs in the paragraph. Vascular smooth muscle cells (VSMCs).**

Once again, we want to thank you for your valuable feedback and for taking the time to review our manuscript. Your input has helped to improve the quality of our work, and we appreciate your efforts in this regard.

Sincerely,

Han Wang, PhD

National Institutes for Food and Drug Control

No. 31 Huatuo Road, Beijing

Email: wanghan926926@126.com
