# Supplementary material for: Preparation and in vivo effectiveness evaluation of heparin-loaded PLGA@PCL core-shell fiber small-diameter vascular grafts
Source: PLoS One. 2026 Jan 16;21(1):e0337192. doi: 10.1371/journal.pone.0337192 (PMC12810837; doi:10.1371/journal.pone.0337192)
Supplement: Attachment — Submitted filename: response letter to reviewers (new).docx [file pone.0337192.s003.docx]

Dear Reviewer，

Thank you for your insightful comments and suggestions on our manuscript. We appreciate the time you have taken to provide feedback, and we have carefully considered each of your points. Our responses are provided below:

**Reviewer #1: In general, results from animal experiments without control experiments are unacceptable in a scientific paper. The results using the grafts made with pure PCL in rabbits should be compared. Previous data using other animal species cannot be used as controls.**

**Response：**Thanks for your conductive suggestions. We reapplied 4 more rabbits to verify pure PCL grafts in rabbit carotid artery grafting model. And the results of patency and livability were shown in Figure S1. Moreover, the regeneration of new tissue was characterized by tissue staining and immunohistochemistry.
**Reviewer #2:** **The previous publication information should be properly linked to the current manuscript to strengthen the claim of continuation of the study.**
**Response：**Thanks for your conductive suggestions. We have linked our previous publication to this paper in the part of “Discussion.”

Once again, we want to thank you for your valuable feedback and for taking the time to review our manuscript. Your input has helped to improve the quality of our work, and we appreciate your efforts in this regard.

Sincerely,

Han Wang, PhD

National Institutes for Food and Drug Control

No. 31 Huatuo Road, Beijing

Email: wanghan926926@126.com
