# Supplementary material for: Preparation and in vivo effectiveness evaluation of heparin-loaded PLGA@PCL core-shell fiber small-diameter vascular grafts
Source: PLoS One. 2026 Jan 16;21(1):e0337192. doi: 10.1371/journal.pone.0337192 (PMC12810837; doi:10.1371/journal.pone.0337192)
Supplement: Attachment — Submitted filename: response letter to reviewers.docx [file pone.0337192.s005.docx]

Dear Reviewer，

Thank you for your insightful comments and suggestions on our manuscript. We appreciate the time you have taken to provide feedback, and we have carefully considered each of your points. Our responses are provided below:

**1.****I f the reviewer comments include a recommendation to cite specific previously published works, please review and evaluate these publications to determine whether they are relevant and should be cited. There is no requirement to cite these works unless the editor has indicated otherwise.**

**Response：**Thanks for your conductive suggestions. This article cites the literature previously published by my research team, mainly References 3, 9, 20, and 21. Among them, the vascular preparation methods involved in references 20 and 21 have great reference value for this paper. In this study, similar methods to those in previous studies were applied in the material preparation process, so References 20 and 21 have not been modified. However, after evaluation, we believed that references 3 and 9 could be deleted. We removed them and replaced them with other references.

**2. Please review your reference list to ensure that it is complete and correct. If you have cited papers that have been retracted, please include the rationale for doing so in the manuscript text, or remove these references and replace them with relevant current references. Any changes to the reference list should be mentioned in the rebuttal letter that accompanies your revised manuscript. If you need to cite a retracted article, indicate the article’s retracted status in the References list and also include a citation and full reference for the retraction notice.**
**Response：**Thanks for your conductive suggestions. The citation format of reference 7 is incomplete. We have made corresponding modifications. Reference 10 was previously a quoted web address. However, a latest reference has now been published, so this new reference has replaced the previous one.

**3.The supplementary Figure S1 is not provided with the revised manuscript and the reapplication ethical clearance certificate should have been provided.**

**Response：**We have supplemented Figure S1 in the article. Currently, it is numbered Figure 5 in the article. In addition, we have uploaded the new ethical license certificate.

**4.Other modifications**

This work was supported by the National Key R&D Program of China (2022YFC2409802). We have added information on financial support in the article

In the previous version, the affiliated institutions of some authors were filled in incorrectly. We have made corrections

Once again, we want to thank you for your valuable feedback and for taking the time to review our manuscript. Your input has helped to improve the quality of our work, and we appreciate your efforts in this regard.

Sincerely,

Han Wang, PhD

National Institutes for Food and Drug Control

No. 31 Huatuo Road, Beijing

Email: wanghan926926@126.com
